# Cone-beam CT evaluation of impacted mandibular third molars and their possible association with mandibular incisor crowding

**DOI:** 10.4317/medoral.27823

**Published:** 2025-11-22

**Authors:** Melda Pelin Akkitap

**Affiliations:** 1 Department of Oral and Maxillofacial Radiology, Faculty of Dentistry, Biruni University, Istanbul, Turkey

## Abstract

**Background:**

The relationship between impacted mandibular third molars and mandibular incisor crowding remains controversial. This study aimed to evaluate whether the impaction pattern of mandibular third molars is associated with lower incisor crowding using cone-beam computed tomography (CBCT).

**Material and Methods:**

A retrospective sample of 140 patients was analyzed, including 70 with unilateral and 70 with bilateral mandibular third molar impactions. Little's Irregularity Index (LII) was measured on CBCT images to quantify incisor crowding. Arch length, depth, intercanine width, and intermolar width were also recorded. Non-parametric tests were used to compare groups, and intra-observer repeatability was assessed.

**Results:**

The mean LII was 9.04.8, with 85% of patients showing some degree of crowding. Bilateral impaction cases presented significantly higher LII scores than unilateral cases (p=0.047). However, no significant differences were found in arch dimensions between groups, and LII was not associated with gender or age. Categorical analysis of crowding prevalence did not differ significantly between unilateral and bilateral groups.

**Conclusions:**

Bilateral mandibular third molar impaction showed a weak association with greater mandibular incisor irregularity. Crowding is a multifactorial condition, and CBCT may provide additional insight into its assessment in patients with impacted third molars.

## Introduction

Mandibular third molars typically erupt between the ages of 18 and 24, yet they frequently fail to fully emerge into the dental arch and may remain partially or completely impacted (1). The potential role of impacted third molars in the etiology of mandibular incisor crowding has been debated for decades (2 - 4). Some authors suggest that mesially directed forces from impacted third molars contribute to anterior dental crowding, whereas others contend that these forces are insufficient to produce clinically meaningful displacement (5 - 7). The absence of a clear causal relationship has fueled ongoing controversy regarding the need for prophylactic third molar removal to prevent late incisor crowding (8 , 9). Despite the lack of consensus, this topic remains clinically relevant in orthodontic and oral and maxillofacial surgery practice (4 - 10). While some clinicians advocate early third molar removal as a preventive measure against anterior crowding, others emphasize the lack of high-quality evidence supporting this approach (5 - 7). In contemporary orthodontics, noninvasive retention methods-such as fixed or thermoplastic retainers-are widely employed to manage post-treatment relapse (6 - 11). Nevertheless, whether third molars exert a measurable influence on mandibular incisor crowding, and whether their removal offers preventive benefit, remains uncertain.

Previous investigations have predominantly relied on two-dimensional imaging modalities such as orthopantomograms and lateral cephalograms, and often used cast-based analytical tools including Little's Irregularity Index (LII) (3 , 4 , 8 , 19). Although cone-beam computed tomography (CBCT) provides submillimeter, three-dimensional imaging with minimal distortion and overlap, relatively few studies have applied CBCT to this topic (10 - 16). Furthermore, most CBCT-based research has excluded impacted third molars, focusing instead on erupted or extracted teeth (10 - 16).

Given these limitations, the present study aims to assess the potential association between impacted mandibular third molars and mandibular incisor crowding using CBCT. Leveraging the anatomical accuracy of three-dimensional imaging, this investigation seeks to provide clearer insight into a long-standing clinical question.

## Material and Methods

This retrospective radiological study was approved by the Research Ethics Committee of Biruni University Faculty of Dentistry (protocol no. 2024-BAEK/07-62). CBCT datasets were obtained from 140 patients who had been referred for clinical indications related to third molars (such as surgical planning or suspected pathology) between January and December 2024. Scans were not performed for research purposes alone, and all procedures adhered to the ALARA principle.

Patients were classified into two groups according to impaction status: Unilateral (n=70) and bilateral mandibular third molar impactions (n=70). Inclusion criteria were age 25 years, a complete mandibular dental arch excluding third molars, absence of previous orthodontic treatment, intact mandibular anterior teeth without restorations, and CBCT images of diagnostic quality. Exclusion criteria included pathological lesions in the mandible, history of orthognathic surgery, developmental anomalies or syndromes, parafunctional habits, previous third molar extraction, or inadequate imaging.

The effect of impacted mandibular third molars on mandibular incisor crowding was evaluated separately for each case. Based on sagittal plane CBCT images, third molars were classified as soft tissue, partial bony, or full bony impactions according to the degree of coverage observed. All teeth meeting these inclusion criteria were enrolled in the study and categorized according to unilateral or bilateral impaction status (20).

**Figure 1:**
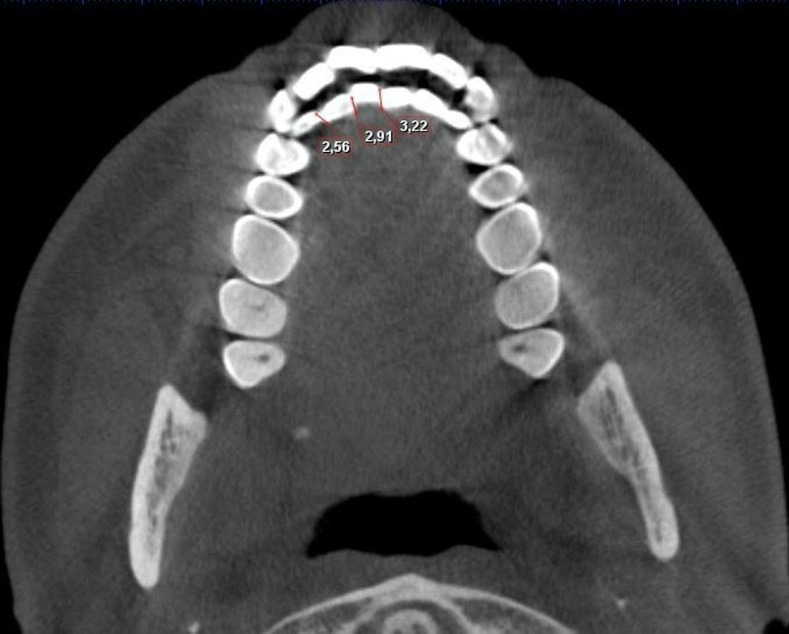
CBCT axial view showing the measurement of LII.

Mandibular incisor crowding was assessed on axial CBCT slices, oriented to minimize visibility of incisal edges and contact points. Crowding was quantified using Little's Irregularity Index (LII) (21), calculated as the sum of linear distances between the mesial incisal edge of each mandibular anterior tooth and the distal incisal edge of its adjacent tooth, extending from the left to the right mandibular canine (Figure 1).

1

Severity was categorized as 0-0.9mm (perfect alignment), 1-3.9mm (minimal), 4-6.9mm (moderate), 7-9.9mm (severe), and &gt;10mm (very severe). For statistical purposes, LII values were dichotomized as "no crowding" (0-3.9mm) or "crowding" (&gt;3.9mm) in accordance with established orthodontic criteria (3 , 10 , 16 , 21).

**Figure 2:**
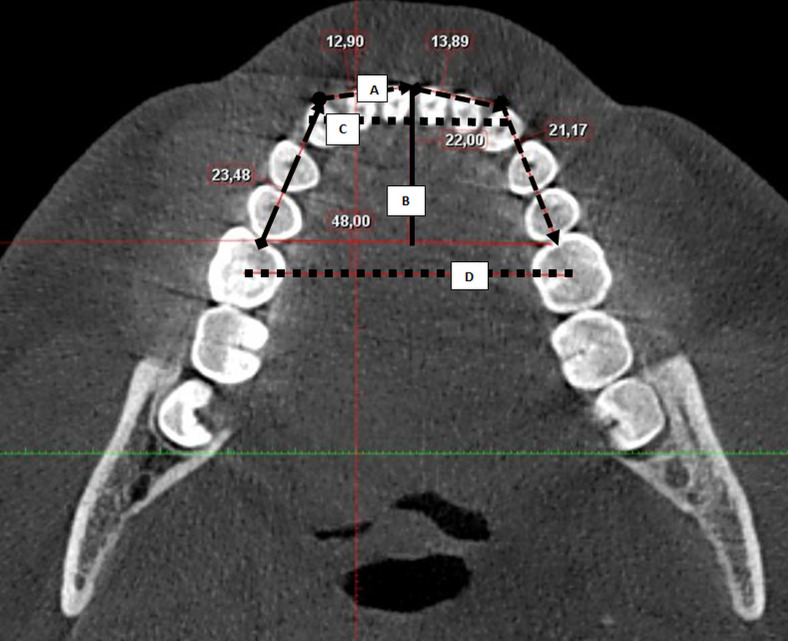
Mandibular dental arch dimensions: (A) arch length, (B) arch depth, (C) inter-canine width, and (D) inter-molar width.

Mandibular arch dimensions were measured in the axial plane, including arch length (sum of anterior and posterior segments mesial to the first molars), arch depth (perpendicular distance from the labial surface of the lower central incisors to the mesial surface of the first molars at the midline), inter-canine width (distance between mandibular canine cusp tips), and inter-molar width (distance between the central fossae of the mandibular first molars) (Figure 2) (22).

2

CBCT scans were acquired using a Sirona Galileos Comfort Plus unit (Dentsply Sirona, York, PA, USA) with patients in a standing position. Imaging parameters included 98 kVp, 6 mA, 36-second rotation time, voxel size 250m, and a 150150mm field of view. Images were evaluated with Sidexis software (Dentsply Sirona). All measurements were performed by an experienced oral and maxillofacial radiologist with more than seven years of clinical experience. To assess intra-observer reliability, 30 randomly selected scans were re-evaluated after an 8-week interval, with Cohen's kappa coefficients exceeding 0.90, indicating excellent agreement. Inter-observer reliability was not assessed due to the single-examiner design.

Statistical analysis was performed with SPSS v27.0. Normality was tested with Kolmogorov-Smirnov. Chi-square tests were used for categorical variables, Mann-Whitney U and Kruskal-Wallis for group comparisons, and Spearman's rank correlation for associations between continuous variables. Significance was set at p&lt;0.05. An a priori power analysis (G*Power; version 3.1, Heinrich-Heine-Universitt Dsseldorf, Germany) estimated a minimum of 116 subjects; the final sample (n=140) exceeded this requirement.

## Results

A total of 140 participants were included in the study, with a balanced gender distribution (50.71% females, n=71; 49.29% males, n=69). The mean age was 32.547.4 years. Most participants were aged 30-39 years (n=60, 42.86%), followed by 25-29 years (n=58, 41.43%), with smaller proportions aged 40-49 years (n=16, 11.43%) and 50 years (n=6, 4.29%). Males were slightly older on average than females (33.196.62 years vs. 31.928.07 years), and this difference was statistically significant (p=0.037).

The unilateral impaction group (n=70) showed equal gender distribution (50% female, 50% male) and a mean age of 34.007.71 years. Most were aged 30-39 years (45.71%) or 25-29 years (32.86%). In the bilateral impaction group (n=70), 51.43% were female and 48.57% were male, with a mean age of 31.096.81 years. The majority in this group were aged 25-29 years (50%) and 30-39 years (40%).

Mean mandibular arch measurements for the total sample were: Arch depth 22.524.15mm, inter-canine width 28.972.46mm, inter-molar width 45.713.22mm, and arch length 68.163.51mm. No significant differences were observed between unilateral and bilateral impaction groups for any arch dimension (p&gt;0.05). However, males had significantly greater inter-canine width, inter-molar width, and arch length than females (all p&lt;0.001), with no significant difference in arch depth (p=0.278) (Table 1).

Table 1

The overall mean LII score was 9.004.78. There were no significant differences in LII scores between females and males (p=0.970) or among age groups (p=0.497). However, the bilateral impaction group had significantly higher LII scores than the unilateral group (p=0.047) (Table 2).

Table 2

Regarding crowding severity, 4.29% (n=6) had ideal alignment/no irregularity, 10.71% (n=15) had minimal irregularity, 20% (n=28) had moderate irregularity, 30.71% (n=43) had severe irregularity, and 34.29% (n=48) had very severe irregularity. Overall, 15% (n=21) of participants had no crowding, while 85% (n=119) presented with crowding of varying severity.

LII scores and crowding severity distributions were comparable between genders (p=0.814 for severity grades; p=0.758 for crowding presence) and across age groups (p=0.970 for scores; p=0.497 for severity grades). Participants aged 50 years tended to have a higher proportion of very severe irregularity (Table 3).

Table 3

Arch measurements did not differ significantly across LII severity categories (p&gt;0.05) or between participants with and without crowding (p&gt;0.05) (Table 4).

Table 4

Similarly, the distribution of LII severity grades (p=0.235) and crowding presence (p=0.098) did not significantly differ between unilateral and bilateral impaction groups (Table 5).

Table 5

## Discussion

The relationship between impacted mandibular third molars and anterior crowding has long been debated. Some authors have suggested that mesial forces from impacted third molars may contribute to incisor crowding (2 , 3 , 12), while others failed to demonstrate a significant association (17). In the present study, bilateral impactions were associated with slightly higher mean LII scores than unilateral impactions, although this effect was modest and of borderline statistical significance (p=0.047).

Despite the statistically significant difference in mean LII values, categorical analyses of crowding prevalence and severity did not reveal consistent group differences. This apparent discrepancy may reflect the small magnitude of the effect, the sensitivity of cutoff thresholds, and limited statistical power. Thus, the results suggest only a weak association rather than a robust or causal link.

Anterior crowding is multifactorial and cannot be attributed solely to third molars. Contributing factors include late mandibular growth, tooth size-arch length discrepancy, and soft tissue pressures (5 , 8 , 18 , 19 , 26). The mean LII in this sample was 9.004.78, with 85% of patients exhibiting some degree of crowding, which is consistent with global epidemiological data (23) and findings from other populations (16). No significant differences in LII scores by gender or age were observed, supporting reports that tooth size and arch length play a more decisive role than gender or chronological age (25).

The observation of greater crowding severity in the bilateral impaction group aligns with studies linking bilateral impactions to increased anterior irregularity (2 , 3 , 12). However, in this study, arch dimensions (arch depth, inter-canine width, inter-molar width, and arch length) did not significantly differ between groups, suggesting that skeletal and developmental factors are likely more influential than impaction status alone (21 , 25).

The use of CBCT provided valuable three-dimensional insights into impaction and arch measurements, though standardization of CBCT-based linear parameters remains limited and further validation against intraoral scans or digital models is needed (10 - 16). Importantly, the scans were retrospectively obtained for clinical purposes, in line with the ALARA principle.

Several limitations must be acknowledged. The retrospective design introduces potential selection bias, and the lack of a non-impaction control group limits interpretation. All measurements were performed by a single examiner, so inter-observer reliability was not assessed. Moreover, no multivariable models adjusting for confounders such as age, gender, or arch parameters were applied. Consequently, residual confounding cannot be excluded. Finally, while the observed difference in LII reached statistical significance, its clinical relevance remains uncertain.

## Conclusions

Bilateral mandibular third molar impactions were associated with slightly greater mandibular incisor irregularity compared with unilateral impactions, but the effect was small and inconsistent across analyses. Given the multifactorial etiology of anterior crowding, these findings should not be interpreted as causal evidence. While CBCT offers valuable diagnostic information for surgical planning, the results do not justify routine prophylactic extraction of asymptomatic third molars. Prospective studies with larger samples, control groups, and adjusted statistical models are needed to clarify these associations.

## Figures and Tables

**Table 1:** Table Arch measurements by impaction status, age group, and gender.

Category	Arch Depth (mm)	Inter-Canine Width (mm)	Inter-Molar Width (mm)	Arch Length (mm)
Impaction Status				
Unilateral	22.785.43	29.122.81	45.572.91	67.923.37
Bilateral	22.262.28	28.822.05	45.853.52	68.413.65
p-value	0.953	0.815	0.546	0.561
Age Group (years)				
20-29	22.542.32	28.882.98	44.863.10	68.523.69
30-39	22.585.85	29.112.01	46.583.44	67.923.38
40-49	22.721.54	29.062.18	45.281.92	68.133.23
50	21.272.25	28.171.89	46.343.03	67.244.25
p-value	0.246	0.616	0.063	0.725
Gender				
Female	22.665.41	28.061.94	44.573.11	67.093.13
Male	22.382.25	29.902.59	46.882.92	69.263.56
p-value	0.278	<0.001	<0.001	<0.001

p-values calculated using Mann-Whitney U test and Kruskal-Wallis test.

**Table 2:** Table Comparison of LII scores by impaction status, age group and gender.

Category	MeanSD	Median (Min-Max)	p-value
Impaction Status			
Unilateral	8.194.73	7.82 (0-23.47)	0.047
Bilateral	9.804.73	8.44 (0-19.87)	
Age Group (years)			
20-29	8.914.62	8.13 (0-19.87)	0.497
30-39	8.824.55	8.34 (0-21.57)	
40-49	9.126.53	7.88 (0-23.47)	
50 yrs	11.333.59	11.57 (5.63-16.31)	
Gender			
Female	8.844.19	8.29 (0-19.35)	0.970
Male	9.165.34	8.16 (0-23.47)	

SD: Standard deviation. Min-Max: Minimum-Maximum. p-values calculated using Mann-Whitney U test and Kruskal-Wallis test.

**Table 3:** Table Distribution of LII grades and crowding status by age group and gender.

Variable	20-29 (n,%)	30-39 (n,%)	40-49 (n,%)	50 (n,%)	p-value	Female (n, %)	Male (n, %)	p-value
LII Grade								
Ideal / No irregularity	2 (3.45)	2 (3.33)	2 (12.50)	0 (0.00)	0.814	2 (2.82)	4 (5.80)	0.814
Minimal irregularity	5 (8.62)	8 (13.33)	2 (12.50)	0 (0.00)		8 (11.27)	7 (10.14)	
Moderate irregularity	15 (25.86)	10 (16.67)	2 (12.50)	1 (16.67)		16 (22.54)	12 (17.39)	
Severe irregularity	17 (29.31)	21 (35.00)	4 (25.00)	1 (16.67)		20 (28.17)	23 (33.33)	
Very severe irregularity	19 (32.76)	19 (31.67)	6 (37.50)	4 (66.67)		25 (35.21)	23 (33.33)	
Crowding Status								
No crowding	7 (12.07)	10 (16.67)	4 (25.00)	0 (0.00)	0.758	10 (14.08)	11 (15.94)	0.758
Crowding present	51 (87.93)	50 (83.33)	12 (75.00)	6 (100.00)		61 (85.92)	58 (84.06)	

LII: Littles Irregularity Index. n: number. p-values calculated using Kruskal-Wallis test and Chi-square test.

**Table 4:** Table Arch measurements according to LII grades and crowding status.

Measurement	Statistic	Ideal / No Irregularity	Minimal Irregularity	Moderate Irregularity	Severe Irregularity	Very Severe Irregularity	p-value	No Crowding	Crowding Present	p-value
Arch Depth (mm)	MeanSD	21.872.69	22.451.40	22.081.83	22.856.97	22.582.01	0.729	22.291.80	22.564.45	0.926
	Median(Min-Max)	22.4(16.8-24.8)	22.4(19.6-25.2)	22.8(18.4-25.1)	22.0(11.6-64.5)	22.61(17.62-28.4)		22.4(16.8-25.2)	22.4(11.6-64.5)	
Inter-Canine Width (mm)	MeanSD	29.092.45	29.462.08	28.481.99	29.323.11	28.772.16	0.521	29.352.13	28.902.51	0.370
	Median(Min-Max)	29.82(25.6-32.41)	29.0(26.8-33.61)	28.62(24.4-33.4)	29.2(22.8-43.6)	28.8(24.43-35.22)		29.64(25.6-33.61)	28.8(22.8-43.6)	
Inter-Molar Width (mm)	MeanSD	44.402.25	45.392.93	45.122.92	46.603.44	45.523.30	0.180	45.112.74	45.813.30	0.221
	Median(Min-Max)	44.61(41.2-47.2)	44.41(42.01-52.81)	44.8(39.2-54.4)	46.4(40.0-54.29)	45.63(37.4-54.8)		44.41(41.2-52.81)	45.6(37.4-54.8)	
Arch Length (mm)	MeanSD	67.664.05	68.793.59	67.903.12	67.653.28	68.633.87	0.807	68.473.66	68.113.50	0.905
	Median(Min-Max)	67.41(62.64-73.82)	67.21(63.51-75.62)	68.16(60.37-72.27)	67.53(59.82-73.73)	68.32(60.89-79.21)		67.21(62.64-75.62)	68.12(59.82-79.21)	

LII: Littles Irregularity Index. n: Number. SD: Standard deviation. Min-Max: Minimum-Maximum. p-values calculated using Kruskal-Wallis test and Mann-Whitney U test.

**Table 5:** Table Distribution of LII grades and crowding status according to unilateral and bilateral groups.

Variable	Unilateral (n, %)	Bilateral (n, %)	p-value
LII Grade			
Ideal / No irregularity	5 (7.14)	1 (1.43)	0.235
Minimal irregularity	9 (12.86)	6 (8.57)	
Moderate irregularity	14 (20.00)	14 (20.00)	
Severe irregularity	23 (32.86)	20 (28.57)	
Very severe irregularity	19 (27.14)	29 (41.43)	
Crowding Status			
No crowding	14 (20.00)	7 (10.00)	0.098
Crowding present	56 (80.00)	63 (90.00)	

LII: Littles Irregularity Index. n: Number. p-values calculated using Chi-square test.

## Data Availability

Declared none.
